# miR-939, as an important regulator in various cancers pathogenesis, has diagnostic, prognostic, and therapeutic values: a review

**DOI:** 10.1186/s43046-024-00220-8

**Published:** 2024-04-29

**Authors:** Hosein Kouchaki, Parnia Kamyab, Farzaneh Darbeheshti, Arezou Gharezade, Hamed Fouladseresht, Reza Tabrizi

**Affiliations:** 1 Shiraz Institute for Cancer Research, School of Medicine, Shiraz University of Medical Sciences, Shiraz, Iran; 2 USERN Office, Fasa University of Medical Sciences, Fasa, Iran; 3 Department of Radiation Oncology, Dana Farber Cancer Institute and Brigham and Women’s Hospital, Harvard Medical School, Boston, MA USA; 4 Department of Immunology, School of Medicine, Isfahan University of Medical Sciences, Isfahan, Iran; 5 Clinical Research Development Unit, Valiasr Hospital, Fasa University of Medical Sciences, Fasa, Iran; 6 Noncommunicable Diseases Research Center, Fasa University of Medical Science, Fasa, Iran

**Keywords:** miR-939, Cancer, Diagnostic, Prognostic, Therapeutic

## Abstract

**Background:**

MicroRNAs (miRNAs or miRs) are highly conserved non-coding RNAs with a short length (18–24 nucleotides) that directly bind to a complementary sequence within 3′-untranslated regions of their target mRNAs and regulate gene expression, post-transcriptionally. They play crucial roles in diverse biological processes, including cell proliferation, apoptosis, and differentiation. In the context of cancer, miRNAs are key regulators of growth, angiogenesis, metastasis, and drug resistance.

**Main body:**

This review primarily focuses on miR-939 and its expanding roles and target genes in cancer pathogenesis. It compiles findings from various investigations. MiRNAs, due to their dysregulated expression in tumor environments, hold potential as cancer biomarkers. Several studies have highlighted the dysregulation of miR-939 expression in human cancers.

**Conclusion:**

Our study highlights the potential of miR-939 as a valuable target in cancer diagnosis, prognosis, and treatment. The aberrant expression of miR-939, along with other miRNAs, underscores their significance in advancing our understanding of cancer biology and their promise in personalized cancer care.

## Introduction

Despite the advanced diagnosis, monitoring, and management methods, the mortality rate due to various cancers is still high, as cases are diagnosed at the late disease stages. Hence, access to biomarkers that can provide valuable information about disease status is urgently needed for managing cancers.

**Fig. 1:**
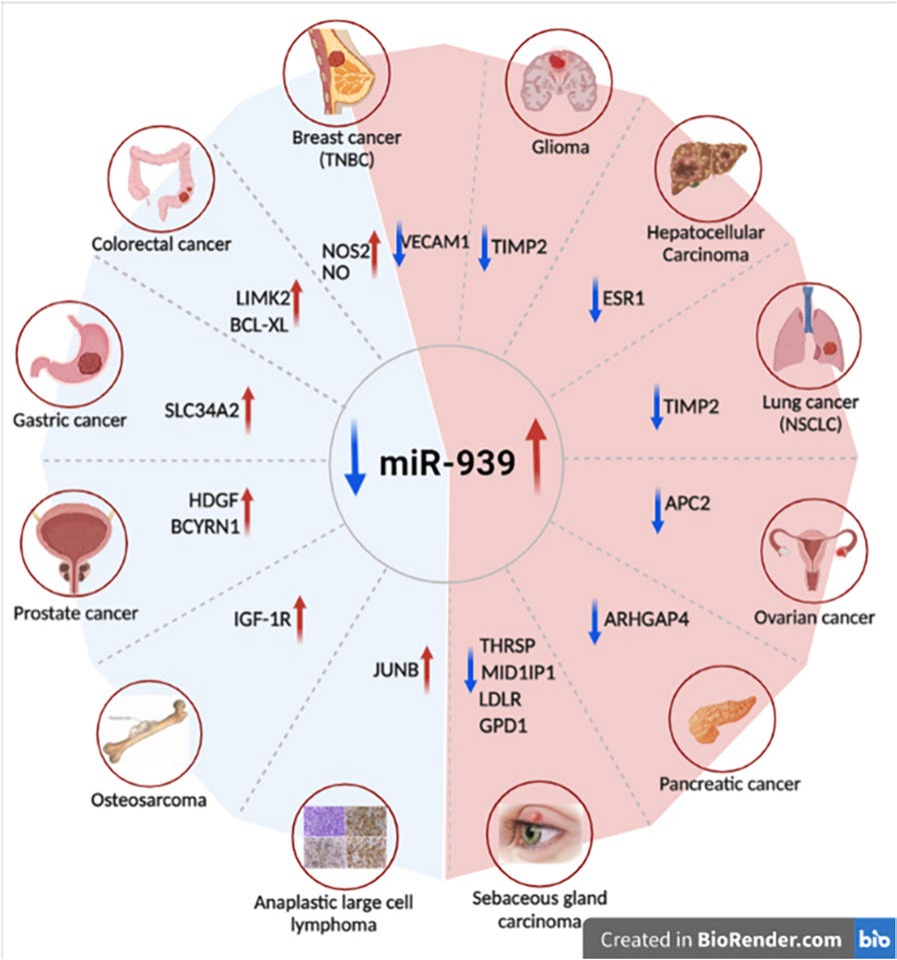
The role of miR-939 in various cancers. Dysregulation of miR-939 reveals its oncogenic (red background) or suppressive (blue background) role in various cancers. Abbreviations: APC2, APC regulator of WNT signaling pathway 2; ARHGAP4, Rho GTPase‐activating protein 4; BCL-XL, B cell lymphoma-extra large; BCYRN1, brain cytoplasmic RNA 1; ESR1, estrogen receptor 1; GPD1, glycerol-3-phosphate dehydrogenase 1; HDGF, hepatoma-derived growth factor; IGF-1R, insulin-like growth factor type 1 receptor; JUNB, JunB proto-oncogene; LDLR, low-density lipoprotein receptor; LIMK2, LIM domain kinase 2; MID1IP1, MID1 interacting protein 1; miR, microRNAs; NO, nitric oxide; NOS2, nitric oxide synthase 2; NSCLC, non-small cell lung cancer; SLC34A2, solute carrier family 34 member 2; THRSP, thyroid hormone responsive spot 14; TIMP2, tissue inhibitor of metalloproteinases 2; TNBC, triple‐negative breast cancer; VE-cadherin, vascular endothelial-cadherin; ↓, decrease; ↑, increase

MicroRNAs (also called miRNAs or miRs) are short and endogenous non-coding RNAs that range in size from 18 to 24 nucleotides. Due to their crucial roles in several cellular functions and almost all biological processes, they have been introduced as promising biomarkers in different cancer types [1, 2]. It is estimated that the expression of 30% of human genes is controlled by miRNAs [3]. MiRNAs do not have any role in encoding proteins independently. However, they recognize the 3′-untranslated regions (UTR) of messenger RNA (mRNA) via a complementary 6–8 nucleotide sequence, affect mRNA stability, and inhibit protein translation processes [4, 5]. Thus, miRNAs are associated with cellular homeostasis pathways such as proliferation, differentiation, and apoptosis by crosstalking with mRNA expression [2, 6, 7]. Due to the undeniable effects of miRNAs on various human biological processes, their close relation to various cancers is not out of mind [8]. Several studies have shown miRNA dysregulation in cancer cells and tumor microenvironments [9]. The protective and promoting effects of miRNAs in different cancers could appear when they inhibit the expression of oncogenes and tumor suppressor genes, respectively (Fig. 1) [1, 10]. Besides, miRNAs play essential roles in regulating anti-tumor responses of the immune system by controlling immune checkpoints [11–13].

A widely reported miRNA, miR-939, has gained significant attention recently due to its unique role in the development of various malignancies, including hepatocellular carcinoma [14], gastric [15], ovarian [16], lung [17], colorectal [18], pancreatic [19], and prostate [20] cancers. Numerous studies have emphasized the remarkable role of miR-939 in the oncogenic process as indicated by its association with advanced stages, altered cell proliferation, high invasion, and metastatic potential [17, 19–22]. In the current study, the pathogenic mechanisms of miR-939 in various cancers have been discussed in detail. We have also reviewed the diagnostic, prognostic, and therapeutic values of miR-939 in association with other molecules and genes involved in the pathogenesis of cancers.

## MiRNA-939 and various cancers

### Pancreatic cancer

Pancreatic cancer is the 12th most common and the 6th most lethal cancer, according to GLOBOCAN 2020 [23, 24]. The high metastatic capacity and poor prognosis of pancreatic tumors have introduced them as a critical global burden [25]. The 5-year survival rate of patients with pancreatic cancer in the American population is only 10%, and 80–85% of cases are incurable. It is predicted that its burden will keep increasing and will become the second etiology of cancer mortality in the USA during the next 20 to 30 years [26, 27]. Therefore, recognizing new molecular targets involved in the progression of pancreatic cancer is essential to improve diagnosis, prognosis, and managing disease, and decreasing mortality.

Recent studies have indicated the role of Rho GTPase-activating proteins (RhoGAPs), such as Rho GTPase‐activating protein 4 (ARHGAP4), as tumor repressors in human cancers, particularly pancreatic cancer, where they play an important role in angiogenesis, aggressive behaviors, staging, and outcomes of tumors [28, 29]. In this regard, miR-939 negatively regulates the expression of ARHGAP4 by binding to the 3′-UTR of the mRNA [19]. Because of the anti-tumorgenesis role of ARHGAP4, positive impacts of miR-939 are expected in the progression of pancreatic neoplasms. The results of a study have shown elevated levels of miR-939 in tumor tissues compared to normal mucosa. Also, they have reported positive correlations between the expression levels of miR-939 and poor prognosis and a low survival rate in patients with pancreatic cancer. Besides, the role of miR-939 in cell migration and invasion has been confirmed by stimulating pancreatic cells with miR-939 and comparing them with the control group [19].

### Hepatocellular carcinoma

Globally, tumors originating from hepatic tissue are among the six most prevalent cancers and the fourth cause of malignancy-related mortality [30]. More than three-fourths of primary liver cancers appear as hepatocellular carcinoma (HCC), which severely burdens healthcare organizations [31]. Although clear improvements have been made in diagnosing, monitoring, and managing cancers, new cases and deaths related to HCC are still growing worldwide due to late diagnosis [30]. The prognosis of HCC is closely dependent on the diagnostic, monitoring, and management procedures [32]. Identifying and targeting effective molecules in disease progression could significantly reduce the HCC burden.

Following this goal, we investigate miR-939’s role as a potential diagnostic and therapeutic target in HCC based on previous reports. Findings have reported a higher level of miR-939 expression in HCC tissue compared to healthy adjuvant mucosa. It is also found that low expression of miR-939 is associated with a good prognosis and high survival rate in HCC patients [21]. Chen et al. employed an imitator or inhibitor to investigate the role of miR-939 in HCC progression. The miR-939 inhibitor suppressed the translation of epithelial-mesenchymal transition (EMT)-related proteins in LM3 cells, while the miR-939 imitator induced the cell invasion. In terms of the mechanism of action, miR-939 downregulates the estrogen receptor 1 (ESR1) expression, an HCC tumor suppressor gene, by modulating protein functions in the cytoplasm and gene expression in the nucleus [21, 33, 34]. It has been shown that the elevated level of ESR1 expression in patients with HCC leads to better disease outcomes. Downregulation of ESR1, on the other hand, is linked to increased EMT and invasiveness of LM3 cells [21]. Besides, the correlation between miR-939 and HCC has been substantiated in another study. Fornari et al. reported that the level of miR-939 expression in HCC-positive cirrhotic patients is significantly higher compared to HCC-negative cases [14]. Overall, focusing on the miR-939 role would be an innovative strategy for managing HCC.

### Prostate cancer

Following lung and colorectal tumors, prostate malignancies are in third place among the most diagnosed cancers worldwide (7.3% of all patients) [23]. They are the most frequently diagnosed cancers in European and USA male populations [35]. In recent decades, significant development has occurred in the early detection and treatment of prostate neoplasms. However, hoped-for improvements in the long-term prognosis have yet to materialize [36, 37]. Although several tumor-suppressing and activating genes that play antithetical roles in disease progression have been identified, the molecular pathways contributing to disease progression are not entirely understood [38, 39]. To date, prostate-specific antigen (PSA) serum level is the primary biomarker for diagnosing prostate cancer. Nonetheless, due to low specificity and limitations in early disease detection, researchers have recently focused on discovering new biomarkers [40, 41].

One of the novel molecules is miR-939, which has been shown to play a protective role against various aspects of prostate tumorigenesis [20, 42]. It is reported that the expression of miR-939 in prostate tumor specimens is regulated lower than in non-malignant prostate tissue [20]. Besides, a meta-analysis that studied the expression level of 37 miRNAs suggested lower expression of miR-939 in recurrent prostate cancer samples compared to non-recurrent ones [43]. Also, it has been found that silencing the expression of miR-939 is closely associated with poor outcomes and lower survival in patients with prostate cancer. Conversely, the proliferation of prostate cancer cells is suppressed by the overexpression of miR-939 [20]. The upregulation of miR-939 in prostate malignant tissue is correlated with decreased prefiltration capacity, colony constitution, migration and invasion, and enhanced apoptosis of tumor cells in vitro [20]. Inversely, hepatoma-derived growth factor (HDGF) is expressed at a higher level in malignant cell lines and directly confronts the antineoplastic activity of miR-939. Bioinformatics analysis and luciferase reporter assays have confirmed the direct binding of miR-939 to the 3′-UTR of the HDGF gene, which subsequently downregulates the expression of HDGF-related mRNAs and proteins [20]. The results of another study demonstrated that the overexpression of brain cytoplasmic RNA 1 (BCYRN1) is related to poor prognosis and the metastatic ability of prostate malignant cell lines by downregulating miR-939 expression [42]. Overall, these findings reveal the diagnostic and prognostic roles of miR-939 in prostate tumors.

### Glioma

Gliomas are CNS neoplasms that originate from the brain and spinal cord glial cells and have the most frequent malignant picture among all central nervous system cancers in young adults [44]. According to the classification published by the World Health Organization (WHO), 2nd-grade and 3rd-grade gliomas are classified as diffuse lower-grade gliomas (LGGs), and 4th-grade gliomas are characterized as glioblastomas (GBMs) [45]. High-grade gliomas, such as GBM and anaplastic astrocytoma, are responsible for both adult and pediatric glioma-related deaths [46, 47]. The global incidence rate of glioma has significantly grown in recent years [48]. Meanwhile, GBM is the most common type of glioma (57.7%), diagnosed in 3.23 individuals out of 100,000 Americans [49].

Surgery is still the main strategy for treatment, despite the deployment of new methods in the management of glioma patients, such as radiotherapy, chemotherapy, and immunotherapy [50, 51]. Although there are combinations of diagnostic and therapeutic procedures for glioma, none have a good prognosis and do not reduce disease recurrence [52, 53]. Thus, discovering novel molecular targets with the capacity to become prognostic and therapeutic biomarkers is one of the urgent needs in glioma management.

MiRNAs are potential targets for this purpose due to their regulatory role in cancer. Findings have shown the upregulation of miR-939 in glioma cell lines [22]. Besides, some glioma characteristics, including tumor size and grading (based on the WHO definition), are significantly correlated with miR-939 expression. Also, proliferation, colony constitution, invasion, and migration of malignant cells are observable in miR-939 overexpression [22]. As a result, the findings suggested that miR-939 plays tumorigenic roles in the pathophysiology of gliomas and is associated with poor outcomes [22]. Bioinformatic analysis and luciferase assay have confirmed that tissue inhibitor of metalloproteinases 2 (TIMP2), introduced as a tumor inhibitor gene in glioma, is a target gene for miR-939. MiR-939 binds to the 3′-UTR of TIMP2 mRNA and reduces its translation [22]. Hence, miR-939 could be a prognostic and therapeutic biomarker in glioma.

### Lung cancer

Lung tumors are the leading cause of cancer-related mortality (18% of total deaths) and the second most common cancer worldwide [23]. Nearly 85% of lung malignancies belong to the non-small cell lung cancer (NSCLC) subgroup, accounting for most lung cancer deaths [54, 55]. NSCLC consists of three subtypes: adenocarcinoma (ADC), squamous cell carcinoma (SCC), and large cell carcinoma (LCC) [56]. Despite the molecular and histological dissimilarity of NSCLC subtypes, the treatment strategy was almost similar until now [57]. However, recent clinical trials suggest different methods for managing NSCLC subtypes to meet the best goals and reduce side effects [57, 58]. On the other hand, small cell lung cancer (SCLC), which accounts for only 15% of all lung tumor cases, is more malignant and invasive and has a lower survival rate than NSCLC [59, 60]. Despite the development of new lung cancer treatment methods, the 5-year survival rate remains less than 10% mainly because of the late diagnosis [61]. Therefore, access to helpful biomarkers for diagnosing, monitoring, and managing patients with lung cancer would improve long-term outcomes.

Recently, miR-939 was reported as an oncogenic micro-RNA in lung cancers [17, 62, 63]. A higher level of miR-939 expression has been identified in lung cancer tissues than in normal lung mucosa [17]. Also, a comparative study between lung cancer patients with high and low expression levels of miR-939 has shown a positive correlation of this miRNA with metastatic abilities and advanced tumor grading (TNM stages) [17]. This study revealed that transfection of miR-939 emulators into tumor cells stimulated cell proliferation, migration, and invasion. On the other hand, NSCLC cell lines showed a repressed proliferative capacity after miR-939 inhibitor transfection [62]. Furthermore, miR-939 is an independent poor prognostic factor in lung cancer patients, whereas its low expression is related to higher survival [17].

In ADC, the most frequent subtype of NSCLC responsible for more than half of all lung cancer cases [56], miR-939 could be an early diagnostic factor [64]. It is reported that miR-939 is positively regulated in most patients with ADC compared to the same sex and age control group, whereas miR-939 expression levels change with each ADC stage. Its expression decreases in stage 3 compared to stages 1 and 2 and rises again at stage 4 [63]. The results of a study indicated the regulatory role of miR-939 on TIMP Metallopeptidase Inhibitor 2 (TIMP2) expression in lung cancer. Accordingly, TIMP2, the tissue inhibitor of matrix metalloproteinase (MMPs), is an NSCLC-related gene that correlates with the expression level of miR-939. MiR-939 regulates TIMP2 expression at the translational level by binding to the 3′-UTR of TIMP2 mRNA [62]. The findings suggest that miR-939 plays an important role in lung cancer and its subtypes. Hence, miR-939 may be a potential target in early detection, predicting outcomes, and treating lung cancer. Further studies are needed to evaluate its function, accurately.

### Ovarian cancer

Ovarian cancer (OC) is the fifth most frequent cause of mortality among women's malignancies and accounts for the most annual deaths of gynecologic cancers in developed societies [65]. Epithelial ovarian cancer (EOC) is the most common OCs, with about a 90% incidence rate [65]. Despite significant progress in therapeutic modalities, OC is diagnosed in an advanced stage (stages III-IV according to the International Federation of Gynecology and Obstetrics (FIGO) staging system), with a 5-year survival rate of less than 30% [66]. This late detection results from poor screening methods [66]. Hence, recognizing efficacious diagnostic and prognostic factors is essential in OC.

Ying and colleagues found that the expression of miR-939 in OC cell lines is regulated at a higher level compared to normal adjuvant ovarian tissue [67]. Also, it has been shown that proliferative, colony-forming, and cell growth abilities enhance after the transfection of OC cells with miR-939 mimics. Thus, miR-939 positively regulates tumorigenesis in OC and can be one of the novel biomarkers for diagnoses and management. Studies confirmed a negative association between miR-939 expression levels and APC2, a tumor suppressor gene, via suppression of the Wnt/ β-catenin signaling pathway [67]. Activation of the wnt/β-catenin pathway modulates extensive cell proliferation and differentiation [68–70] via upregulating cyclin D1, c-MYC, and TCF genes in several human cancers [71–75]. Consequently, miR-939 overexpression, as an oncogene, leads to more protein production from the cyclin D1 and c-MYC genes [67]. Accordingly, a luciferase reporter assay has demonstrated that miR-939 binds the 3′-UTR site of the APC2 mRNA and suppresses its translation. On the other hand, inhibiting miR-939 has a positive effect on APC2 protein translation [67].

The miR-939 also acts as a link between platelet microparticles (PMPs) and EMT. A study has shown that the expression level of miR-939 is elevated in the PMPs. Also, a correlation is reported between miR-939 and the expression level of EMT-associated molecules, including vimentin, E-cadherin, and claudin [16]. PMPs are small components derived from the stimulation of platelets with thrombin and play a crucial role in OC oncogenicity by boosting tumor cells' proliferation and migration capability. In addition, PMPs induce metastatic behaviors (cell proliferation and migration) in the EOC via modulating EMT-related molecules [16]. In this regard, miR-939 stimulates OC development and metastatic behaviors by stimulating vimentin expression and inhibiting endothelial (E)-cadherin and claudin expression [76, 77]. It is reported that miR-939 could be a predictive biomarker for choosing the most efficient treatment strategy. By tissue biopsy or using ascites, miR-939 expression levels could be measured, and the proper decision about immediate surgery would be made [78]. Overall, miR-939 might be a potential target for the diagnosis, monitoring, and treatment of ovarian cancer.

### Colorectal cancer

Colorectal cancer (CRC), with a 1.9 million incidence rate and a 0.9 million mortality rate in 2020, is placed in the 3rd rank of the most frequent cancers and the 2nd rank of the most fatal cancers worldwide. Global statistics in 2020 showed that CRC was responsible for 10% and 9.4% of all new cancer cases and cancer-associated mortality, respectively. The CRC burden is predicted to keep increasing in further years and reach 3.2 million new patients in 2040. This growth rate results from the industrialization of human lifestyles and the developing and aging population [79, 80]. Although early CRC diagnosis plays a fundamental role in suppressing metastasis and limiting mortality and morbidity, unfortunately, CRC manifestation is revealed only in the late stages. Because of this, more than half of CRC patients die due to long-distance metastasis, particularly metastasis to liver tissue [81]. Thus, introducing novel biomarkers to relieve the CRC burden is urgent.

MiR-939 can be a molecular target for this purpose, and its role is reported in CRC progression. Zhang et al. discovered suppressor functions for miR-939 in CRC. In this regard, there was an inverse association between miR-939 levels and long intergenic non-protein coding RNA 460 (LINC00460) for regulating LIM domain kinase 2 (LIMK2) expression. Whereas miR-939 suppresses the expression of LIMK2 mRNA via binding to its specific sequence, LIN00460 upregulates the expression of LIMK2 by sponging miR-939. LIMK2 promotes the migration and invasion of cancer cells and the poor prognosis of CRC. Hence, miR-939 and LIN00460 act as suppressors and oncogenes in CRC pathogenesis, respectively [18]. Long noncoding RNA HEIH (LncRNA-HEIH) is another miR-939-related oncogenic LncRNA that promotes the viability, proliferation, and invasion of CRC cells by inducing B cell lymphoma-extra large (Bcl-xL) expression. This LncRNA acts as a sponge for miR-939 and competes with the organizing nuclear factor-κB (NF-κB)-miR-939 complex. Following this, NF-κB targets the Bcl-xL promoter and upregulates its expression [82].

In colon cancer (stage T2-3N0), a study showed a negative correlation between the miR-939 level and “shorter distant metastasis-free survival (DMFS)” [83]. Another study did not demonstrate a significant difference in the expression levels of miR-939 between colon adenocarcinoma patients responding and non-responding to chemotherapy [84]. Therefore, miR-939 has no diagnostic value for determining chemoresistant colon cancer cases [84], whereas it has a prognostic value in the early stages of colon cancer [83].

### Gastric cancer

In 2020, gastric cancer (GC) accounted for 5.6% (6th rank) of global cancer incidence and 7.7% (3rd rank) of cancer mortality [23]. In different regions, the incidence rate varies by up to 15–20-fold [85]. For example, GC is not among the ten most common cancers by occurrence or death in the US population [86]. But eastern randomized trials elucidated 30 to 40% higher GC surveillance compared to Western ones [87, 88]. Up until now, the primary strategies for treatment have been surgical resection and palliative chemotherapy [89]. The prognosis of GC patients is still unfavorable despite the implications of novel therapeutic approaches, and chemotherapy resistance and metastatic behaviors are the main etiologies of mortality in patients. Nevertheless, GC-related molecular pathways are not clearly understood [90–92]. Hence, investigating key molecules related to GC would be critical in determining potential targets for early diagnosis and management of the disease.

A study found a drastically reduced expression level of miR-939 in GC tissues and cell lines compared to normal tissues and cell lines. Besides, a study of patients with stage I-III GC cancer found that local relapse or distant metastasis is associated with a decrease in miR-939 expression in GC tissue [15]. Hence, miR-939 has been introduced as a prognostic biomarker for predicting GC patients’ responses to chemotherapy. Findings show that complete or partial response to therapy in GC patients is associated with upregulation of miR-939, whereas downregulation of miR-939 is common in the no response and progressive groups [15]. Accordingly, a novel therapeutic strategy is applying miR-939 and 5-fluorouracil (Fu) simultaneously, which have a synergic effect on the apoptosis or necrosis of GC cells and inhibit clonogenicity. In the first 48 h, miR-939 sensitizes GC cells to 5-Fu, a commonly used chemotherapeutic agent in GC patients. In addition, study findings showed that miR-939 silencing enhanced oncogenesis and cell proliferation of GC cell lines in vitro [15].

Regarding the mechanism of action, solute carrier family 34 member 2 (SLC34A2) is a direct target gene for miR-939 that negatively correlates with it [15]. Upregulation of miR-939 significantly downregulates expression of SLC34A2 mRNA by binding to the 3′-UTR site and inhibits MEK1/2 phosphorylation and Raf-1 level, resulting in the suppression of proliferation, migration, and invasion in cells [93]. The elevated expression of miR-939 in patients with GC improves survival and reduces tumor recurrence rate. While SLC34A2 upregulation is associated with unsatisfactory survival and increased tumor recurrence. GC patients with an elevated level of miR-939 and a reduced level of SLC34A2 present the best outcomes and survival rates [15]. Overall, miR-939 could be an oncosuppressor biomarker with promising prognostic and therapeutic value in patients with GC.

### Breast cancer

Breast cancer (BC) is the most common form of cancer worldwide [23] and the first cause of death among all cancers in women 20 to 50 years old [65]. BC mortality rate varies greatly from the East to Europe and the USA [94]. This difference results from various environmental factors and lifestyles, which have been demonstrated by increased mortality and incidence rates during migration from safe BC regions to areas with higher mortality rates [95]. Metastasis and relapse, as the primary cause of malignancy-associated death, occur in 10 to 20% of the European population with BC after initial relief [96, 97]. The most aggressive BC subtype, triple‐negative breast cancer (TNBC), comprises 15–20% of all BC cases. The negative expression of three receptors, including the estrogen receptor (ER), progesterone receptor (PR), and human epidermal growth factor receptor-2 (HER2), is the unique specification of TNBC [98, 99]. The first incidental rank of BC among all tumors, recurrences, and metastatic behaviors emphasizes the necessity of recognizing potential molecular targets for early diagnosis, monitoring, and effective therapy.

In this regard, the expression of miR-939 in BC cells is regulated at a higher level than in healthy breast tissues, and it is a frequent finding in the highly invasive BC subtype (TNBC) [100]. Furthermore, it has been discovered that the synergistic effects of miR-939 upregulation and lymph node involvement increase the risk of TNBC relapse by approximately sixfold. More findings have shown that miR-939 exerts a significant role in TNBC migration [100] through blood vessels instead of lymphatic metastasis [101]. In silico analysis determined that miR-939 targets vascular endothelial (VE)-cadherin mRNA (3UTR), which regulates the junction between endothelial cells and controls vessel permeability. In confirmation, miR-939 directly reduced 40 to 60% of VE-cadherin protein levels in HUVEC, but no changes in the level of VE-cadherin mRNA have been seen. This suggests that miR-939 inhibits the translation of VE-cadherin mRNA instead of mRNA degradation. A decline in VE-cadherin protein levels after miR-939 overexpression weakens the monolayer integrity of endothelial cells in the vascular system. As a result, it opens some "gates" to malignant cells in blood vessels that facilitate hematogenous metastasis. Hence, elevating miR-939 in TNBC is correlated with poor disease-free survival [100].

Interestingly, a recent study has suggested the downregulation of miR-939 in all BC subtypes (including TNBC) compared to non-malignant mammary controls [102]. In this study, nitric oxide synthase (NOS)2/NOS3 expression and, as a result, high NO production (an immunosuppressor and cancer inducer) were negatively correlated with miR-939 expression in TNBC cells. These findings suggested that miR‐939 is a tumor suppressor in TNBC by hindering the NOS2/NO axis. Transfection of miR-939 into MDA‐MB‐231 cells (a type of TNBC cell line) significantly depresses NOS2-induced NO production, but not NOS3. Besides, miR-939 upregulation in TNBC tissues induced cell apoptosis and attenuated cell migration and colony formation. Recently, it has been found that lncRNA HEIH leads to tumor progression by increasing the expression of NOS2, PDL1, and MICA/B and decreasing the expression of NKG2D ligands in cancer cells. The sponging of miR-939 has been introduced as a probable mechanism for the lncRNA HEIH acts in TNBC [102]. Overall, these paradoxical results express the indigence of further studies about the role of miR-939 in BC and its subtypes.

### Osteosarcoma

Osteosarcoma (OS), the most frequent bone malignancy, is more prevalent among children and adolescents [103]. Long bone metaphysis, including the distal femur (43%), proximal tibia (23%), and proximal humor (10%), are the major origins of OS [104]. To our knowledge, OS's annual incidence and mortality rate have developed over recent years [105, 106]. Combining systemic chemotherapy and extensive surgical resection is the most helpful therapeutic technique [107–109], which has resulted in a 60–70% improvement in the 5-year survival rate of patients with non-metastatic osteosarcoma [110]. However, the long-term outcome is still unsatisfactory, and this multistage therapy could survive only 11–30% of patients with distant metastasis [111, 112]. Hence, improving the prognosis of OS patients would be impossible unless the pathophysiological pathways underlying OS tumorigenicity were understood.

MiR-939 is one of the novel biomarkers whose role has been determined in OS pathophysiology. Zhao et al. reported a decreased expression level of miR-939 in OS tissue compared to paired adjacent tissue. They also indicated that OS patients with a low level of miR-939 present advanced clinical stages and more distant metastases than patients with a higher level. An in vitro study confirmed that invasive abilities in OS are associated with miR-939 expression level [113]. In this regard, the proliferation, viability, metastatic, and migratory behaviors of cancer cells were suppressed by transfection and overexpression of miR-939. MiR-939 was also transfected into nude mice to determine its impact in vivo. Results indicated that the tumor weight and size were inversely related to the expression level of miR-939 [113]. In addition, miR-939 injection into tumor xenografts downregulated the expression of insulin-like growth factor type 1 receptor (IGF-1R), p-PI3K, and p-Akt compared with the control group. Bioinformatic analysis and luciferase assays introduce IGF-1R as a direct target gene for miR-939, which is highly expressed in OS cell lines [113]. MiR-939 binds to the IGF-1R gene’s 3′-UTR sequence and inhibits IGF-1R-associated mRNA expression and protein translation. The results indicate that the overexpression of IGF-1R increases proliferation and migration and decreases apoptosis of cancer cells in OS samples by activating the PI3K/Akt axis [113]. Hence, miR-939 has a prognostic value in osteosarcoma cases, and its downregulation is associated with a poor prognosis and a reduced overall survival rate.

### Sebaceous gland carcinoma of the eyelid

Sebaceous gland carcinoma (SGC) is a malignant tumor type of the sebaceous gland that occurs in the eyelid. SGC placed in the 2nd and 3rd (or 4th) rank of the most prevalent malignant tumors arising from the eyelid in some Asian and European countries, respectively [114, 115]. Two main therapeutic strategies are extensive resection and elective radiotherapy. However, delayed disease diagnosis decreases these therapies' impacts and enhances morbidity and mortality in patients with SGC [116]. Meanwhile, late diagnosis of SGC is correlated with aggressive behaviors such as invasion into lymph nodes and other organs, leading to a nearly 6–9% mortality rate [117–119] and attenuating long-term prognosis and survival [116]. Therefore, determining molecular targets for early diagnosis and more effective treatment of SCG is urgently needed to improve patient prognosis.

The leading etiology of SGC development is abnormal lipid metabolism and accumulation in the cytoplasm of the meibomian and Zeis glands, the primary sites of SGC initiation [120]; hence, investigating lipid metabolism pathways can provide good clues about effective molecules in the SGC. It has been shown that the upregulation of miR-939 (plus 15 other miRNAs) followed by the downregulation of 516 mRNAs is associated with the loss of lipid metabolism function and is enriched in cholesterol biosynthesis pathways. Therefore, miR-939 can be a potential diagnostic and therapeutic target.

Thyroid hormone responsive spot 14 (THRSP), MID1 interacting protein 1 (MID1IP1), low-density lipoprotein receptor (LDLR), and glycerol-3-phosphate dehydrogenase 1 (GPD1), which control fatty acid, cholesterol, and triglyceride synthesis, are all linked to miR-939 expression. Overall, miR-939 is involved in tumorigenesis of the sebaceous gland through its role in ectopic lipid metabolism [120]. However, the results mentioned above were derived from bioinformatics analysis. Further molecular research is warranted to substantiate the existing evidence.

### Pediatric anaplastic large cell lymphoma

One rare subtype of peripheral T cell lymphoma is anaplastic large cell lymphoma (ALCL), which is more common among children and young adults than in older patients [121]. Based on the WHO categorization, ALCL consists of two subgroups, ALCL with upregulation of anaplastic lymphoma kinase (ALK^+^ ALCL) and ALCL with downregulation of ALK expression (ALK^−^ ALCL) [122]. Most pediatrics with ALCL are ALK^+^ (90%), which is characterized by t(2;5)(p23;q35) chromosomal translocation and overexpression of nucleophosmin/ALK (NPM-ALK) fusion protein [123]. ALK^+^ ALCL reveals more aggression and growth abilities compared to the ALK^−^ ALCL subgroup. Consequently, the relapse of the ALK^+^ ALCL is approximately sixfold in comparison with ALK^−^ ALCL patients (5 months versus 30 months) [123]. It is shown that ALK expression and its signaling cascade are associated with the ALCL carcinogenic pathways and pathogenesis [124–126]. To determine the molecular pathways correlated with ALK^+^ ALCL invasive behavior, various miRNAs were introduced as potential targets. The differences in miR-939 levels have been reported between ALK-positive and ALK-negative cell lines. The results have also suggested a notable upregulation of miR-939 in ALK-negative tumor cells. Moreover, it has been demonstrated that miR-939 up-modulation prominently inhibits the invasion, migration, clonogenicity, and growth capacity of entire ALCL malignant cells. However, miR-939 has shown no control over NPM-ALK expression. Regarding the mechanism of action, JUNB is a target gene for miR-939 in ALK^+^ ALCL cell lines, associated with increased cell proliferation. MiR-939 binds to the 3′-UTR of JUNB mRNA and regulates JUNB-related protein levels. Platelet-derived growth factor receptor B (PDGFRB) is a direct transcriptional target for JUNB, contributing to ALCL oncogenicity and lymphomagenesis. PDGFR inhibitors, when combined with ALK, decrease the relapse rates of ALCL cases [127]. Finally, the findings introduced miR-939 as a tumor-suppressor gene in ALCL via suppression of the JUNB/PDGFRB axis.

## Prospects and challenges

MiR-939 has immense influence over a range of cellular functions, not only in the normal metabolism of healthy cells but also in the tumorigenic process of malignant tissues. The accumulated results reveal that miR-939 is a critical regulator of cellular processes such as proliferation, differentiation, development, and death, especially in cancer. Although we mentioned the role of miR-939 in the pathogenicity of many malignancies, the following challenges remain to be solved in further investigations. (1) Only 12 variant types of cancer have been evaluated to date, and the precise function of miR-939 in many other cancers remains unclear. (2) Additionally, among these 12 types, certain cancers such as glioma require further evaluation and the provision of stronger evidence. (3) Our evidence about this microRNA, compared to the older ones, needs to be revised, and there are still several fields to be investigated. (4) The cellular interaction between miR-939 and other molecular pathways needs more clarity in different cancer types. (5) Regulating the expression level of miR-939 could be a consequence or a cause of oncogenesis; hence, finding the exact fact requires more evaluation.

## Conclusion

In the final word, we provided a comprehensive review of the miR-939 dysregulation in twelve types of cancer, as summarized in Table 1. By targeting various signaling pathways and processes, miR-939 plays roles in tumor cell proliferation or growth, EMT, apoptosis rate, invasive or metastatic capacities, and resistance to therapeutic methods. Hence, miR-939 can be a potential target for cancer identification, patient surveillance, and long-term outcomes. Numerous investigations exploring the association between miR-939 and tumor figures have identified either tumor suppressor or promoter effects of miR-939 in cancers. Despite the revelation of predictor diagnostic and prognostic value of miR-939 in recent studies, we sincerely hope this review provides more passion for further investigations with larger sample sizes about the clinical potential of miR-939 in cancers.

**Table 1:** The expression of miR-939 and target genes in various cancers

Cancers	MiR-939 expression	Role	Target genes expression	Effects on cancer cells	References
Pancreatic cancer	↑	Onc	↓ ARHGAP4	Migration, invasion	[19, 28, 29]
Hepatocellular carcinoma	↑	Onc	↓ ESR1	EMT, invasion	[21, 33, 34]
Prostate cancer	↓	TS	↑ HDGF	Proliferation, viability, colony formation, migration, invasion	[20, 43]
			↑ BCYRN1	Metastasis	[42, 43]
Glioma	↑	Onc	↓ TIMP2	Proliferation, colony formation, migration, invasion	[22]
Lung cancer (NSCLC)	↑	Onc	↓ TIMP2	Proliferation, migration, invasion	[17, 62, 63]
Ovarian cancer	↑	Onc	↓ APC2	Proliferation, colony formation	[67–75]
Colorectal cancer	↓	TS	↑ LIMK2	Migration, invasion	[18, 82]
			↑ BCL-XL	Viability, proliferation, invasion	[82]
Gastric cancer	↓	TS	↑ SLC34A2	Proliferation, migration, invasion	[15, 93]
Breast cancer (TNBC)	↑	Onc	↓ VECAM1	Metastasis	[100, 101]
	↓	TS	↑ NO & NOS2	Viability, colony formation, migration	[102]
Osteosarcoma	↓	TS	↑ IGF-1R	Proliferation, viability, migration invasion	[113]
Sebaceous gland carcinoma of the eyelid	↑	Onc	↓ THRSP, ↓ MID1IP1, ↓ LDLR, ↓ GPD1	Abnormal metabolism, accumulation of lipids in the cytoplasm of the meibomian and Zeis glands	[120]
Pediatric anaplastic large cell lymphoma	↓	TS	↑ JUNB	Proliferation, migration, invasive, clonogenicity	[127]

*Abbreviations*: *APC2* APC regulator of WNT signaling pathway 2, *ARHGAP4* Rho GTPase‐activating protein 4, *BCL-XL* B cell lymphoma-extra large, *BCYRN1* Brain cytoplasmic RNA 1, *EMT* Epithelial‑mesenchymal transition, *ESR1* Estrogen receptor 1, *GPD1* Glycerol-3-phosphate dehydrogenase 1, *HDGF* Hepatoma-derived growth factor, *IGF-1R* Insulin-like growth factor type 1 receptor, *JUNB* JunB proto-oncogene, *LDLR* Low-density lipoprotein receptor, *LIMK2* LIM domain kinase 2, *MID1IP1* MID1 interacting protein 1, *miR* microRNAs, *NO* Nitric oxide, *NOS2* Nitric oxide synthase 2, *NSCLC* Non-small cell lung cancer, *Onc* Oncogenic, *SLC34A2* Solute carrier family 34 member 2, *THRSP* Thyroid hormone responsive spot 14, *TIMP2* Tissue inhibitor of metalloproteinases 2, *TNBC* Triple‐negative breast cancer, I tumor suppressor, *VE-cadherin* vascular endothelial-cadherin, ↓ Decrease, ↑ Increase

## Data Availability

Not applicable.

## Abbreviations

ADC: Adenocarcinoma
Akt: AKT serine/threonine kinase 1
ALCL: Anaplastic large cell lymphoma
ALK: Anaplastic lymphoma kinase
APC2: APC regulator of WNT signaling pathway 2
ARHGAP4: Rho GTPase‐activating protein 4
BC: Breast cancer
BCL-XL: B cell lymphoma-extra large
BCYRN1: Brain cytoplasmic RNA 1
c-MYC: MYC proto-oncogene
CRC: Colorectal cancer
DMFS: Distant metastasis-free survival
E-cadherin: Endothelial-cadherin
EMT: Epithelial‑mesenchymal transition
EOC: Epithelial ovarian cancer
ER: Estrogen receptor
ESR1: Estrogen receptor 1
5-FU: 5-Fluorouracil
GBM: Glioblastoma
GC: Gastric cancer
GPD1: Glycerol-3-phosphate dehydrogenase 1
HCC: Hepatocellular carcinoma
HDGF: Hepatoma-derived growth factor
HER2: Human epidermal growth factor receptor-2
IGF-1R: Insulin-like growth factor type 1 receptor
JUNB: JunB proto-oncogene
LCC: Large cell carcinoma
LDLR: Low-density lipoprotein receptor
LGG: Lower-grade glioma
LIMK2: LIM domain kinase 2
LINC00460: Long intergenic non-protein coding RNA 460
LncRNA-HEIH: Hepatocellular carcinoma upregulated EZH2-associated long non-coding RNA
MEK: Mitogen-activated protein kinase
MID1IP1: MID1 interacting protein 1
miR: MicroRNAs
MMPs: Metalloproteinase
mRNA: Messenger RNA
NF-κB: Nuclear factor-κB
NO: Nitric oxide
NOS2: Nitric oxide synthase 2
NSCLC: Non-small cell lung cancer
OC: Ovarian cancer
Onc: Oncogenic
OS: Osteosarcoma
PI3K: Phosphatidylinositol 3-kinase
PMP: Platelet microparticles
PR: Progesterone receptor
PSA: Prostate-specific antigen
Raf-1: Raf-1 proto-oncogene (serine/threonine kinase)
SCC: Squamous cell carcinoma
SCLC: Small cell lung cancer
SGC: Sebaceous gland carcinoma
SLC34A2: Solute carrier family 34 member 2
THRSP: Thyroid hormone responsive spot 14
TIMP2: Tissue inhibitor of metalloproteinases 2
TNBC: Triple‐negative breast cancer
TS: Tumor suppressor
UTR: Untranslated region
VE-cadherin: Vascular endothelial-cadherin
WHO: World Health Organization
